# Understanding the Role of Irisin in Longevity and Aging: A Narrative Review

**DOI:** 10.3390/epidemiologia6010001

**Published:** 2025-01-08

**Authors:** Ana I. Plácido, Daniela Azevedo, Maria Teresa Herdeiro, Manuel Morgado, Fátima Roque

**Affiliations:** 1 Biotechnology Research, Innovation and Design for Health Products (BRIDGES), Research Laboratory on Epidemiology and Population Health, Polytechnic of Guarda Av. Dr. Francisco Sá Carneiro 50, 6300-559 Guarda, Portugal; dazevedo@ipg.pt (D.A.); froque@ipg.pt (F.R.); 2 Department of Medical Sciences, Institute of Biomedicine (iBiMED), University of Aveiro, 3810-193 Aveiro, Portugal; teresaherdeiro@ua.pt; 3 Health Sciences Research Centre, University of Beira Interior (CICS-UBI), 6200-506 Covilhã, Portugal; mmorgado@fcsaude.ubi.pt; 4 Pharmaceutical Services of Local Health Unit of Cova da Beira (ULS Cova da Beira), 6200-251 Covilhã, Portugal; 5 Faculty of Health Sciences, University of Beira Interior (FCS-UBI), 6200-506 Covilhã, Portugal

**Keywords:** irisin, aging-related diseases, older adults, longevity

## Abstract

Irisin is a protein resulting from a proteolytic cleavage of fibronectin type III domain-containing protein 5 (FND5). The ability of irisin to modulate adipocyte and control glucose metabolism in human metabolic diseases gave rise to the hypothesis that irisin could have a pivotal role in aging-related diseases. Although in animal models, increased levels of irisin have been positively associated with better health outcomes, in humans, its role remains controversial. To provide an overview of the main finding on irisin in older adults, a comprehensive search was performed through the MEDLINE-PubMed, Web of Science, Scopus, and Cochrane databases for studies conducted in older adults (≥60 years) published since 2012. After grouping and analyzing the articles based on diseases associated with older adults, the main conclusion of this narrative review is that the included studies did not yield consistent evidence regarding the association between irisin and health or disease in older adults. Further studies are necessary to clarify the effective role of this protein in promoting health and longevity.

## 1. Introduction

In 2021, 20.8% of European citizens were older adults. According to the projections, this number will reach 30.3% in 2058 [1]. Longer, healthy lives present new challenges and opportunities for older adults, their families, and society. The World Health Organization (WHO) warns that if the increase in life expectancy is not accompanied by an increase in additional years of good health, the sustainability of health services will be compromised [2]. This highlights the need to promote the well-being of older adults. The aging-related decline in physiological function makes older adults more prone to a loss of resilience and increased vulnerability to diseases, such as cardiovascular diseases, cancer, diabetes, hypertension, dementia, and osteoporosis [3,4]. Ensuring the quality of health in the last years of life is crucial, and identifying reliable and robust biomarkers of aging plays a key role in this issue. In recent years, it has been suggested that irisin modulates the aging process and may play a significant role in maintaining health and longevity [5,6]. Irisin was first described in 2012 by Bostrom et al. as a PGC1α-dependent extracellular fragment resulting from the proteolytic cleavage of the ectodomain of the precursor protein FNDC5 (fibronectin domain-containing protein) and is secreted from skeletal muscle into the bloodstream under exercise conditions, inducing a transition of white adipose tissue to adipose tissue with browning morphology [7].

Though it was first described in 2012, the history of irisin began a decade earlier when a group of independent researchers characterized an unknown gene expressed in the heart, brain, skeletal muscles, and other tissues during the embryonic development of mice and also in adult animals [8]. The unknown gene received the name fibronectin type III repeat-containing protein 2 (*FRCP2*) [9]. This gene, later named fibronectin type III domain-containing protein 5 (FNDC5), received attention in 2012 because it was found to be one of the target genes of PGC1-α, a transcriptional activator released by skeletal muscle during exercise. Several studies have demonstrated that irisin can reprogram adipocyte metabolism and control glucose homeostasis in human metabolic diseases and other physical exercise-modifiable diseases. Although some findings are controversial, several researchers have suggested that irisin could have a pivotal role in aging-related diseases.

The gene FNDC5 is known to be expressed in multiple tissues and organs, including the heart, liver, and brain, as well as skeletal muscle [10,11,12,13]. Sequencing of irisin shows that this myokine is 100% conserved between rodents and humans [7].

FNDC5 is a highly conserved gene, and the sequencing of irisin shows that this myokine is 100% conserved between rodents and humans [14]. Although controversial, some studies have shown that irisin can potentially influence the metabolism of adipocytes and regulate glucose levels in metabolic and exercise-related diseases [7,15]. Given that several researchers have pointed out that irisin may play a crucial role in aging-related diseases, this work aims to comprehensively review the key findings associated with a hypothetical role of irisin in aging.

## 2. Methods

The search strategy was designed to identify relevant studies addressing the role of irisin in age-related disorders, provided by studies published in the MEDLINE-PubMed, Web of Science, Scopus, and Cochrane databases and published between 1 January 2012, and 31 December 2023. To identify relevant studies, we used the following keywords and medical subject headings (MeSH): “aged”, “geriatrics”, “Irisin”, and “FNDC5”. Additionally, we manually searched the reference lists of the retrieved articles to ensure that all pertinent studies were identified.

The concept of older adults is multidimensional, encompassing chronological, biological, psychological, and social age. In this work, we adopted the definition of an older adult provided by the United Nations as a person who is over 60 years of age while recognizing the diversity of older adults in terms of functional ability and intrinsic capacities [15,16].

## 3. Results

**Figure 1:**
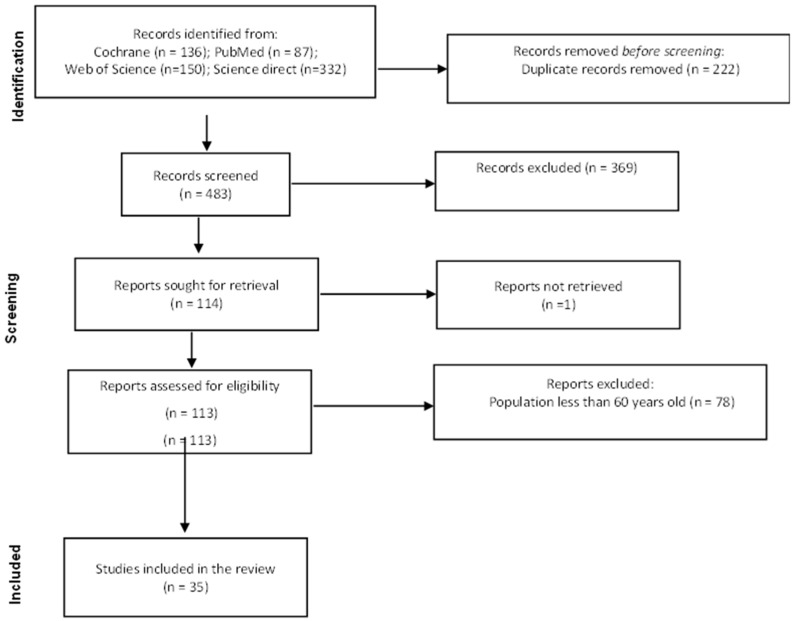
Flow diagram of the literature selection that occurred in this review.

A total of 705 articles were retrieved from the databases, of which 222 were duplicates. After screening by title and abstract, 369 studies did not meet the inclusion criteria and were excluded (did not report levels of irisin, the population was younger than 60 years, or studies were conducted in laboratory models). One hundred and fourteen reports were analyzed in full text, of which 35 were included, and the remaining 76 were excluded because the study population was less than 60 years old (Figure 1). The main characteristics of the included studies are summarized in Table 1.

### 3.1. Serum Irisin Levels in Healthy Older Adults

Even though the reference values for circulating irisin remained undefined, the data reported by different studies suggest that the secretion of irisin is associated with health conditions and increased longevity. During our research, a total of 14 articles [17,18,19,20,21,22,23,24,25,26,27,28,29,51] (Table 1) were found reporting data on irisin in healthy older adults, among which two of those [25,29] conducted were in healthy centenarian older adults.

#### 3.1.1. Irisin in Centenarians

In 2014, Emanuele and colleagues [29] hypothesized that irisin can have a determinant role in the process of successful aging. To demonstrate that, they compared the serum irisin levels of 79 disease-free centenarians, 178 nondiabetic patients who had experienced an acute myocardial infarction, and 180 young, healthy adults. The authors observed that centenarians present statistically significant high levels of irisin (35.3 ± 5.5 ng/mL) when compared to both healthy young controls (20.7 ± 6.3 ng/mL) and patients who had experienced a myocardial infarction (15.1 ± 5.4 ng/mL). After the observation by Staigner et al. [52] that two singe-nucleotide polymorphisms in the FNDC5 gene, rs16835198 and rs726344, were associated with insulin sensitivity, Sanchis-Gomar et al. [25] conducted an analysis of the frequency of FNDC5 single nucleotides polymorphisms (SNPs) rs16835198 and rs726344 among centenarians and disease-free controls of the same ethnic origin (Spanish cohort) as well as in two others that were geographically and ethnically independent cohorts (from Italy and Japan). The authors found no genotype frequency difference between the centenarians and controls in the three cohorts. Furthermore, they were also unable to identify an association between serum irisin levels and the rs726344 and rs16835198 SNPs.

#### 3.1.2. Serum Irisin Levels in Healthy Older Adults with Ongoing Physical Activity

The beneficial effect of physical exercise on health seems to be mediated by various cytokines, including irisin. Several research groups have reported data regarding the role of physical exercise in the expression of irisin and its impact on healthy aging. Our search yielded seven studies [17,18,19,23,26,27,51] reporting data on irisin in healthy older adults undergoing endurance exercise interventions and five studies [20,21,22,24,28] reporting data on irisin in healthy older adults undergoing resistance training. Prestes and colleagues [28] analyzed the irisin serum levels in sedentary women undergoing 16 weeks of resistance training with two weekly sessions. They observed that, overall, the training was not effective in changing the irisin levels or affecting other cytokines such as BDNF, IL1-β, IL10, and IL-15. The authors also observed a variation in irisin values among sedentary women: 7 women presented irisin increments <80th percentile (high responders), 12 had increments between the 50th and 80th percentile (medium responders), and 19 demonstrated irisin increments below the 50th percentile (low responders). In a 16-week, twice-weekly resistance training study, Planella-Farrugia and colleagues [24] found that participants in both groups, the low resistance training group and low resistance training with nutritional support group, showed similar and statistically significant increases in serum irisin levels. In six weeks, twice per week resistance training, Kujawski and colleagues [20] observed that older adults undergoing resistance training had improved cognitive function compared to older adults performing sitting callisthenic balance training. This improvement was not accompanied by significant changes in serum levels of irisin, BDNF, neurotropin 3, and neurotropin 4/5 [20]. In the participants undergoing functional resistance training with or without blood flow restriction, Pazokian et al. observed increases in follistatin and reductions in myostatin, but the levels of irisin were not significantly changed in any of the two groups in comparison to a control group [21].

A study comparing the irisin levels of older adults and younger adults undergoing endurance training, consisting of 3 days per week for 8 weeks, observed that although baseline levels of irisin were higher in young adults than in older adults, after the intervention, young adults did not present significant increases in their irisin levels in comparison to young adults that did not undergo the intervention, and older adults presented increased levels of irisin in comparison to older adults that did not undergo the intervention. The comparison of older and younger adults undergoing intervention revealed that older adults had an increase in serum irisin levels. This increase is associated with a reduction in visceral adipose tissue [27].

In older women undergoing Nordic walking training along with vitamin D supplementation, it was observed that women with higher baseline levels presented an increase in serum irisin levels, while those with lower baseline vitamin D levels exhibited the opposite trend [26]. In both groups, Nordic walking training resulted in a decrease in the autophagy-inducing stress protein HMGB1 and the pleiotropic cytokine IL-6, with small and statistically insignificant changes in BDNF [26]. A significant increase in BDNF levels was also observed in response to regular Nordic walking training. This regular training also improved cognitive functions, accompanied by increased concentrations of irisin and BDNF [18]. A statistically significant increase in the levels of irisin and BDNF were also found in a group of women undergoing a 16-week aquarobic exercise program. In this group of older women, an interaction effect between irisin and BDNF was also observed [51]. Furthermore, an increase in both irisin and BDNF levels was observed in a group of older adults after 10 weeks of tai chi training [19]. These increases in myokines were correlated with improvements in the balance of older adults [19]. To clarify if fitness status influences the secretion of myokines, Biziak et al. [23] performed a pilot study in high-fitness older adults and low-fitness older adults and observed that although the irisin basal levels were higher in high-fitness older adults after an acute exercise, no changes were observed between groups [23]. In a 12-week folk dance training program, it was observed that older females with low, moderate physical activity had improved insulin sensitivity, a significant drop in BDNF, and a rise in irisin concentration [17].

### 3.2. Serum Irisin Levels in Aging-Related Diseases

Several studies, most of them in animal models, have suggested the putative irisin effect on aging-related diseases through improvement in homeostasis metabolism and glucose regulation, less insulin resistance, and decreasing obesity in the control of diabetes and also in the induction of an anti-inflammatory response. Irisin has also been suggested as a potential therapeutic molecule for neurodegenerative disorders. Our search retrieved 22 data on aging-related diseases [22,30,31,32,33,34,35,36,37,38,39,41,42,43,44,45,46,47,48,49,50]. Four studies were conducted in an obese population [22,41,42,43], five in patients with brain disorders [31,32,33,34,41], four studies in chronic obstructive pulmonary diseases [35,36,37,38], five studies in older patients with sarcopenia diseases [35,36,44,45,47], two studies were associated to vascular disease in older adults [48,49], one was related to hip fractures [39], and finally, one study reported data on cancer patients [50].

#### 3.2.1. Serum Irisin Levels in Obese Older Adults

The worldwide prevalence of overweight and obesity has drastically increased in the last decades, and nearly a third of the worldwide population is classified as overweight or obese. Older adults account for the age group with the highest prevalence of obesity. Obesity increases the risk of developing non-communicable diseases, such as coronary health diseases, diabetes, hypertension, and some cancers, and also leads to a low health quality of life and impaired functional ability. Within four studies retrieved by our search, three of them reported data on obese older adults’ ongoing physical activities [22,41,42,43]. Tibana et al. [43] investigated the ability of resistance training to modulate the serum levels of irisin, improve power and force, and reduce fat mass in untrained obese and non-obese women. The authors observed that before a 16-week resistance training program, there was no difference between the serum levels of irisin between groups. However, after the intervention, irisin significantly decreased in the non-obese group and with no changes in the obese group [43]. More recently, a study comparing irisin levels in older obese adults with younger adults in ongoing acute circuit training observed that the irisin levels did not significantly increase under acute circuit training [22]. Finally, a study in obese women with incontinence observed that 3 times weekly sessions of pelvic floor muscle training for 4 weeks led to a significant increase in irisin in obese women with incontinence [41]. A cross-sectional study of 111 obese and 105 non-obese male older adults observed that irisin and leptin concentrations were associated with obesity [42].

#### 3.2.2. Irisin and Brain Diseases

The observations that exercise increases BDNF and irisin, combined with the knowledge that BDNF is a myokine with a relevant role in neuroplasticity and a potential link to learning and memory processes [53], raises the hypothesis that irisin could be associated with cognition. Our search retrieved five studies in which data on irisin serum levels in older adults with brain disorders were reported [30,31,32,33,34]. In 2017, Kuster et al. [34] evaluated the impact of mental and physical exercise training on mild cognitive patients on a set of blood biomarkers, including irisin and BDNF. The authors observed that although mental and physical training did not induce changes in myokines, a positive correlation existed between irisin and BDNF. Additionally, the authors observed a correlation between global cognition and irisin (r = 0.37, *p* = 0.02) and, more specifically, with the component memory function [34]. Also, aiming to assess the impact of physical and mental training on BDNF and irisin levels among older patients with mild cognitive impairment, Damirchi [33] and colleagues randomly assigned 54 sedentary older women into four groups: physical training, mental training, and physical plus mental training and control group. The authors observed a significant increase in BDNF in both the mental training group and the physical exercise plus mental training group. Irisin levels only significantly increased in the combined training group. Interestingly, the physical exercise group had a decreased level of BDNF compared to the control group. Notably, in the mental training group, no correlation was found between irisin and BDNF [33]. In a cohort of 240 older adults with mild cognitive impairment, Lima-filho [30] observed that individuals harboring the FNDC rs1746661 (T) allele had a regional reduction in low glucose metabolism in memory-associated brain regions and an increase in brain amyloid beta PET load. In Alzheimer’s disease patients with behavioral disturbances, it was observed that although the irisin levels remained unchanged in the overall samples, the levels of irisin correlated with the duration of the agitation/aggression states [32]. Mutcher and colleagues [31] evaluated the levels of irisin in depressed-mood older athletes and observed an inverse association between irisin levels and low-grade white matter lesions in the brain, which predicted impaired quality of life.

#### 3.2.3. Serum Irisin Levels in Chronic Obstructive Pulmonary Disease in Older Adults

In older adults, obstructive pulmonary diseases are a common cause of death and illness. This review includes four studies that discuss irisin in chronic obstructive pulmonary disease (COPD) patients [35,36,37,38]; three of them are from Japan [36,37,38], and one is from Brazil [35]. Based on the knowledge that low physical activity has been associated with more frequent hospitalizations in COPD patients and the fact that physical activity levels have been a predictor of mortality in COPD patients, Ijiri et al. [38] hypothesized that irisin could be a biomarker associated with physical activity in COPD patients. They found that COPD patients have lower baseline irisin levels compared to control patients. Additionally, they observed that while acute exercise did not increase serum irisin levels, an 8-week exercise training did increase the irisin levels in COPD patients [38]. In a study with COPD smoker patients, it was found that the irisin serum levels were significantly lower in these patients [37]. The authors also discovered a significant correlation between the serum irisin levels and α-klotho protein, which is speculated to have a circulating anti-aging hormone effect [37]. Decreased levels of irisin were also found by Sugyama et al. in thier sample of COPD patients [36] and by Lage et al. [35] in patients with sarcopenia and COPD.

#### 3.2.4. Serum Irisin Levels in Older Patients with Sarcopenia

Sarcopenia, a neuromuscular disease characterized by a progressive loss of skeletal muscle mass, is a leading cause of loss of health quality in the geriatric population [54]. Sarcopenia is associated with an increased risk of falls and fall-related injuries in older adults [55]. Our search yielded five studies related to irisin in older adults with sarcopenia [35,44,45,46,47]. Within these, three were conducted in Asia [44,45,47], one in Turkey [46], and one in South America [36]. Park et al. [47] took advantage of a cohort of 153 women to investigate the possible role of irisin as a biomarker in sarcopenia and observed that the irisin levels were lower in the sarcopenia group than in the control or pre-sarcopenia groups [47]. Similar results were observed by Alsaawi et al. [44] in a multicenter study conducted to evaluate the prevalence of sarcopenia in older Arab women. Tsai et al. [46] did not find changes in the protein levels of irisin in their sample of sarcopenia older adults. In a cross-sectional study, Baek et al. [46] did not observe a lower concentration of serum irisin according to the status of sarcopenia.

#### 3.2.5. Serum Irisin Levels in Patients with Fractures

The occurrence of fractures is more common as people age, leading to increased morbidity and mortality among older adults; healing potential declines with advanced age, resulting in a greater burden on healthcare services [56]. One study [39] examined the role of irisin in fractures and reported a positive correlation between irisin and bone mineral density in older women, as well as an increased risk of hip fractures in women with low irisin levels [39].

#### 3.2.6. Serum Irisin Levels in Patients with Vascular Diseases

Ischemic stroke is a leading cause of long-term disability in adults and is characterized by insufficient blood flow through cerebral vessels, resulting in cerebral hypoxia, insufficient apport of glucose, and impaired removal of unnecessary metabolites, which results in cerebral infarction. Gaining knowledge and insight into this devasting pathology is essential to reduce the impact of this disease on the well-being of older adults and the impact on healthcare services [56,57]. This review included two studies reporting data on patients’ ischemic stroke. Tu et al. [49] conducted a multicenter study on Chinese patients with ischemic stroke. After stratification of the sample into four quartiles, based on the irisin levels, they observed that patients with poor outcomes (Q1) had irisin levels significantly lower than patients with good outcomes. The authors also found an association between the irisin levels and poor functional outcomes. Over the 6 months follow-up period, the authors observed a mortality rate of 21.2%, with 39.3% associated with the Q1 patients who had low irisin levels and 6.3% in Q4 patients who had high levels of irisin [49]. Bosanack et al. took advantage of fifty-two patients with heart failure with preserved ejection fraction and arterial fibrillation and analyzed the serum levels of irisin, leptin, adiponectin, insulin-like growth factor 1 (IGF-1), and malondialdehyde, and observed that irisin, leptin, and malondialdehyde were significantly lower in these patients [48].

#### 3.2.7. Serum Irisin Levels in Patients with Other Diseases

Over the past century, lifestyle changes have been accompanied by an increase in the prevalence of cancer, and cancer-related deaths have increased over the past decade. Two studies test the hypothesis that irisin has the potential to be a biomarker for early diagnosis of colorectal cancer. Zhu et al. [50] compared serum irisin levels in cancer patients with healthy older adults and observed that patients with high ATF3 and low irisin serum levels were more likely to have cancer [50]. To investigate the potential role of irisin and leptin as markers of diabetes, Sahin-Efe et al. [42] measured the serum levels of irisin in stored blood samples of non-obese, obese, non-obese that, years after, had been diagnosed with diabetes type II, and observed that both leptin and irisin were significantly higher in obese subjects. The authors did not find any correlation between irisin and diabetes prediction.

## 4. Discussion

**Figure 2:**
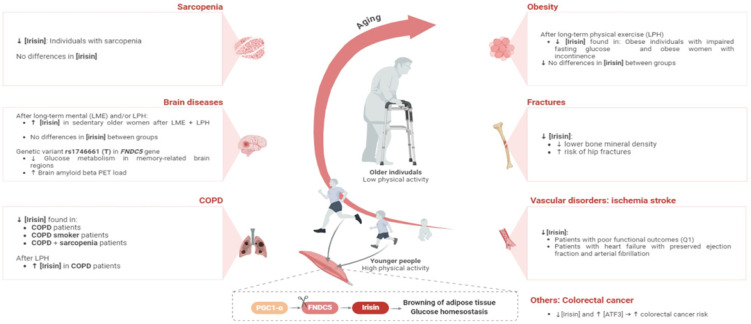
Irisin serum levels according to health condition and/or intervention in older adults.

Since the description of irisin as a myokine secreted by skeletal muscle, whose expression of its precursor, FNDC5, is regulated by PGC1α, a protein produced by exercising muscle [7], irisin has gained attention. Its role in facilitating intercommunication between organs to maintain a healthy state has been studied [56,57]. Presently, it is known that irisin is expressed in organs other than skeletal muscles, including testicles, pancreas, brain, salivary glands, liver, kidney, and heart, in which the expression of irisin is higher than in skeletal muscle, suggesting that irisin may be produced in an autocrine or paracrine way, and reinforcing that it could have a more extensive role beyond being solely a myokine secreted by skeletal muscle [40,58]. It may have a role not only in intercommunication between organs and tissues but also in promoting overall health and longevity. The lack of established reference levels for irisin and the scarcity of studies in aging populations limits the interpretation of the main findings and the ability to perform a critical review of the methods used in the different included studies. The included studies did not provide consistent evidence regarding the association between poor circulating levels of irisin and disease. In Figure 2, we present a synthesis of the evidence provided by the various included studies.

In conditions such as COPD, fractures, vascular diseases, and cancer, lower circulating levels of irisin were observed in older patients. However, in the case of sarcopenia patients, while a recent study suggested that irisin can ameliorate age-associated sarcopenia and metabolic dysfunction [59], one of the included studies failed to find an association between irisin and sarcopenia adults [45]. As previously mentioned, irisin has the potential to promote the browning of white adipose tissue, dissipating energy to produce heat and potentially reducing the appearance of cellulite. Studies conducted in different populations have reported contradictory results. Some authors have suggested a potential relationship between irisin and obesity [42,43], while others have reported conflicting findings [22]. In the included studies, two of them observed that irisin did not increase serum irisin levels. Tibana et al. [43] suggested that changes in body mass composition resulting from resistance training in both obese and non-obese individuals (control group) were not associated with circulating irisin levels. A recent systematic review also indicated that although circulating irisin levels in overweight/obese individuals were higher than in overall healthy controls when ethnicity was considered, higher protein levels were observed in obese/overweight African individuals, while no clear tendency was observed in European, Asian, and American populations [60].

Depression and mild cognitive impairment are risk factors for dementia, and mouse models have suggested that irisin can improve depressive neuropathology through the PGC-1α signaling pathway [12]. In older adults with depressive symptoms, Mutchler et al. [31] also observed an inverse association between irisin and health quality. In cases of mild cognitive impairment, two studies that reported data on patients undergoing physical exercise interventions observed that irisin is correlated with global cognition. However, these studies have shown contradictory results regarding the relationship between irisin and BDNF [33,34].

Under healthy conditions, the data suggest that in individuals with high longevity, centenarian irisin levels are higher than in younger adults or patients with diseases [29]. Although more studies are needed to corroborate the association between irisin and longevity, Rana and colleagues demonstrated that the telomere length is positively correlated with irisin plasma levels and negatively correlated with aging, suggesting a determinant role in the mechanism of telomere length [61]. This effect of irisin on telomere length could be modulated by the peptides Lys-Glu and the neuroprotective peptide Glu-Asp-Arg [62].

Irisin was first associated with myokines secreted by skeletal muscles, leading several researchers to measure its circulating levels after training interventions. Among studies reporting data from resistance training interventions lasting 16 weeks, conducted twice a week in healthy older participants, two reported that overall, the intervention did not increase the levels of irisin, while another study reported that nutritional support alongside the intervention led to an increase in irisin levels. Regarding endurance interventions, all the studies reported increases in irisin levels after the intervention. Gmiat et al. [26] suggested that physical activity combined with vitamin D supplementation is more effective in increasing irisin levels [18,26]. They also suggested that regular practice of physical activity leads to higher secretions of irisin and an increase in BDNF, both of which are associated with better cognition. Another study demonstrated that while participants with better fitness initially had higher levels of irisin than older participants with lower fitness status, there were no significant differences in irisin levels between groups after exercise intervention. This suggests that fitness status may not be a critical factor in the increase in irisin levels [23]. A recent systematic review of randomized control trials suggests that exercise training significantly increases circulating irisin in the overall population [63]. However, in our study, we included studies in which exercise did not increase circulating irisin. We believe that more studies in larger populations are needed to unequivocally demonstrate the role of physical exercise in the modulation of irisin secretion in aging populations.

## 5. Conclusions

Although irisin has shown positive indications in longevity research, further studies are necessary to establish its exact physiological role. To ensure consistent and reliable results, future research should carefully consider various critical factors, including age, lifestyle, diet, and ethnicity. Additionally, analyses should account for comorbidities and concomitant medications. These comprehensive studies will provide a better understanding of irisin’s potential impact on human aging.

## Figures and Tables

**Table 1:** Characteristics of the included studies.

Author, Year, Country	Study Design	Sample Size	Mean Age	Patients-Associated Condition	Irisin			Outcomes
Healthy older adults								
Rodziewicz-Flis et al., 2023 Poland [17]	Randomized control trial	41 Balance training, n = 15 Dance training group, n = 14 Control group, n = 12	71.2 ± 5.5	Disease-free	Balance training group: from 14.5 ± 3.5 to 16.4 ± 4.4 ng/mL; *p* = 0.029 Folk dance training group: from 15.6 ± 4.3 to 17.6 ± 4.5 ng/mL; *p* = 0.022 Control group: unchanged			Folk dance and balance training improved physical performance and blood pressure, accompanied by an increase in irisin levels. The folk dance training group had increased insulin sensitivity.
Gmiat et al., 2018 Poland [18]	Randomized control trial	45 women Beginner group, n = 20 Advanced group, n = 25	68 ± 5.12	Disease-free	Beginners: baseline 12 ± 5 observed change (−22 ± 72%) Advance: baseline 13 ± 8 observed change 4 ± 49%			No direct correlation was noted between vitamin D and cognitive function. The amelioration of cognitive functions may be explained by an increase in irisin and an elevated uptake of tryptophan.
Solianik, et al., 2022 Lithuania [19]	Randomized control study	30 (women) Tai chi, n = 15 Control; n = 15	60–79 years	Disease-free	-			Tai Chi increased irisin levels (*p* < 0.001).
Kujawski et al., 2022 Poland [20]	Two-arm single-blind randomized control trial	69 Sitting callisthenic, n = 31 Resistance training, n = 38	64.6 ± 4 (Resistance training) 67.7 ± 6 (Sitting callisthenic balance)	Disease-free	Resistance group (irisin μg/mL) Before: 19.54 ± 3.3, after 20.46 ± 4 Sitting callisitic group Before: 18.66 ± 4.3; after: 17.63 ± 5.2			Changes in irisin were related to set-shifting and short-term memory.
Pazokian, et al., 2022 Iran [21]	Randomized trial	30 (men) Functional training with blood flow restriction, n = 10 Functional training, n = 10 Control group, n = 10	67.7 ± 5.8	Disease-free	No difference in the level of irisin between the groups ((F = 0.6, *p* = 0.561, η^2^ = 0.04)			No changes in irisin serum levels.
Rioux et al., 2021 Canada [22]	Quasi-experimental randomized trial	26 Older adults, n =13 Younger adults, n = 13	68.00 (64.50–69.50) (Older adults) 24.00 (22.00–30.50) (Younger adults)	Disease-free	No changes			Circuit training did not increase irisin levels.
Bizjak et al., 2021 Germany Iran [23]	Clinical trial (pilot study)	28 Low physical fitness, n =14 High physical fitness, n = 14	75.25 ± 5.44	Disease-free	High physical fitness participants had a higher basal level of irisin than low physical fitness participants (*p* = 0.0195)			Higher basal irisin serum levels in the high physical fitness group revealed slightly beneficial molecular serum and muscle adaptations.
Planella-Farrugia et al., 2019 [24]	Prospective and controlled clinical trial	34 Control group, n = 20 Resistance exercise group, n = 14 Resistance exercise + nutritional support group, n = 9	66.4 ± 4.6 (Control) 64.9 ± 5.5 (Resistance exercise) 71.2 ± 3.3 (Resistance exercise + nutritional support group)	Disease-free	Resistance exercise + nutritional support Baseline: 3 ± 1.1 Follow-up: 2.6 ± 1.3, *p* = 0.030) Resistance training Baseline: 3.1 ± 0.8 Follow-up 2.4 ± 0.3, *p* = 0.011 Control Baseline: 3.1 ± 0.9 Follow-up: 3.5 ± 1.1			Circulating irisin constitutes a marker for improved muscular performance in older adults.
Sanchis-Gomar et al., 2014 Spain [25]	Cohort	2158	100–104 (Italian) 100–116 (Japanese) 100–111 (Spanish)	Disease-free	Genotype frequencies between centenarians and controls Spanish cohort			No differences between genotype/allele frequencies of the two SNPs associated with in vivo insulin sensitivity in centenarians versus controls.
					rs726344 χ^2^ = 2.821, *p* = 0.244; rs16835198 χ^2^ = 1.540, *p* = 0.463	Italian Cohort rs726344 χ^2^ = 0.122, *p* = 0.941; rs16835198 χ^2^ = 1.128, *p* = 0.569	Japanese cohort rs726344- is not present in the cohort rs16835198 χ^2^ = 5.337, *p* < 0.069)	
Kim and Kim, 2018 South Korea [24]	-	26 women Control group, n = 12 Aquarobic exercise group, n = 14	71.43 ± 4.45 (Control) 71.77 ± 3.07 (Aquarobic exercise)	Disease-free	Control group Pre: −165.76 ± 12.53; Post: 157.14 ± 13.97 Aquarobic exercise group: pre: −174.85 ± 11.6; post: 203.62 ± 16.44			Aquarobic exercises increase the serum irisin and BDNF levels.
Gmiat et al., 2017 Poland [26]	-	27	67 ± 8	Disease-free Nordic walking training	Patients with less than 20 ng/mL of vitamin D: Baseline: 11 ± 3 observed changed −23 ± 60% Patients with more than 20 ng/mL of vitamin D Baseline: 10 ± 3 observed changed 5 ± 55			Nordic walking training irisin and improves the uptake of leucine among women with higher baseline vitamin D.
Miyamoto-Mikami et al., 2015 Japan [27]	-	53 Healthy young adults, n = 25 Middle-aged/aged older adults, n = 28	69 ± 6 (Control) 65 ± 8 (Training group)	Disease-free	Control Pre: 142.8 ± 8.7 Post: 144.4 ± 9.2 Training Pre: 140.6 ± 26.7 Post: 140.6 ± 26.7			Secreted irisin may have a role in the exercise-induced alteration of abdominal visceral fat in middle-aged and older adults.
Prestes et al., 2015 Brazil [28]	-	72 (women)	66.90 ± 7.56 (Control) 66.20 ± 6.05 (Linear Periodization group) 65.52 ± 4.72 (Undulating periodization group)	Disease-free	Control: 169.62 ± 36.55 Linear periodization group 230.00 ± 55.88 Undulating periodization group: 202.10 ± 52.30			Although resistance training did not induce a significant effect on body composition and cytokines, the authors identified a group of people that have an increment > 80th percentile (>14.12%) of irisin, suggesting that not all people respond in the same way to physical activity.
Emanuele et al., 2014 [29]	-	Centenarians, n = 79 Patients with precocious acute myocardial infarction, n = 178 Young controls, n = 180	100–104 (Centenarians) 28–39 (Patients with precocious acute myocardial infarction) 27–39 (Young controls)	Disease-free	Centenarians: 35.3 ± 5.5 Patients with precocious acute myocardial infarction: 15.1 ± 5.4 Young controls: 20.7 ± 6.3			Serum irisin is highest in disease-free centenarians compared with young, healthy controls and young patients with myocardial infarction.
Brain diseases								
Lima-Filho et al., 2023 Brazil [30]	Cross-sectional	725 Cognitive unimpaired, n =240 Cognitive impairment, n = 485	73.8 ± 7.37	Cognitive impairment	-			Patients carrying the FNDC5 rs1746661(T) allele presented hypometabolism in Memory-linked brain regions and increased brain amyloid-β PET load.
Mucher et al., 2021 Austria [31]	Cross-sectional study	112 Athletes, n = 56 Controls, n =58	66 (62–68) (Athletes) 66 (63–69) (Controls)	Depression	Irisin [z-score] Athletes: −0.13 [−0.86–0.33] Control: −0.02 [−0.63–0.65] Differences: U = 1359.0; *p* = 0.225			Circulating irisin and the multifunctional cytokine/myokine IL-6 are associated with depressive symptoms among older adults.
Conti et al., 2019 Italy [32]	Cross-sectional study	60 Alzheimer’s disease, n = 40 Control, n = 20	77.6 ± 5.6 (Alzheimer’s disease) 78.7 ± 5.7 (Control)	Alzheimer’s disease	Irisin serum levels were elevated in A/A+ patients (+10.0%; *p* < 0.05)			Irisin is not useful as a surrogate marker for agitation in AD but might represent secondary outcomes when testing drugs for behavioral dysfunction, implying elevated motor activity.
Damirchi, Hosseini, and Babaei, 2018 Iran [33]	Randomized control trial (small scale study)	54 (women) Control group, n = 9 Mental training, n = 15 Physical training, n = 15 Mix training, n = 15	60–85 69.11 ± 4.69 (Control) 67.9 ± 3.75 (Mental training) 68.81± 3.68 (Physical training) 67.76 ± 4.69 (Mix training)	Mild cognitive impairment	Irisin concentration (ng/mL) Control: baseline 13.67 ± 5.23; post-intervention: 12.87 ± 4.95 Physical training: baseline 11.23 ± 2.77 post-intervention: 11.47 ± 3.08 Mental exercise: baseline 10.57 ± 1.99 post-intervention: 9.92 ± 1.66 Physical training + mental exercise; baseline 10.38 ± 1.03			The authors did not observe changes in irisin levels, contradicting a previous study performed by them.
Küster et al., 2017 Germany [34]	Clinical trial	47	71.2 (60–88)	Dementia	Irisin (M) Cognitive training: Baseline 55.2 ± 9.9 Physical training group: Baseline 57.9 ± 10.6 Waitlist group Baseline 56.4 ± 14.1			Irisin and BDNF correlated positively with cognitive function.
COPD								
Lage et al., 2022 Brazil [35]	Cross-sectional study	86 COPD, n = 43 No COPD, n = 43	73.9 (COPD) 72.7 (No COPD)	COPD and sarcopenia	No COPD 1062.8 pg/mL (909.6–1216.2) COPD 904.6 pg/mL (794.3–1014.8)			Plasma irisin levels and inflammation are decreased in older adults with COPD and sarcopenia.
Sugiyama et al., 2017 Japan [36]	-	40	73 ± 9.3	COPD	-			Decreased serum irisin levels are related to emphysema in patients with COPD and are involved in epithelial apoptosis, resulting in emphysema.
Kureya et al., 2016 [37]	-	53 Smokers with COPD, n = 24 Smokers without COPD, n = 13 Non-smokers, n = 16	71 (62–78) (Non-smokers) 66 (62–71) (Smokers without COPD) 70 (65–74) (Smokers with COPD)	COPD	smokers with COPD patients: 26.3 (22.6–32.4) ng/mL; smokers without COPD: 53.7 (46.7–62.8) ng/mL; non-smokers: 58.5 (42.8–78.9) ng/mL			Soluble a-klotho is one possible factor involved in reduced irisin release from skeletal muscle. The disruption of irisin leads to abnormal energy homeostasis in COPD.
Ijiri et al., 2015 Japan [38]	-	99 Control, n = 27 COPD, n = 72	70 (62–75) (Control) 70 (66–74) (COPD)	COPD	COPD patients: 31.6 (22.7–40.4) ng/mL; control subjects: 50.7 (39.3–65.8) ng/mL; *p* < 0.001			Serum irisin level may prove to be a valuable biomarker in clinical follow-up of COPD.
Fractures								
Yan et al., 2018 China [39]	Cross-sectional, case-control study	160 women	70–90	Hip fracture	Cases (361.5 ± 140.0 ng/mL vs. control 478.5 ± 159.6 ng/mL, *p* < 0.001)			Low concentrations of irisin are associated with an increased risk of hip fractures.
Ruan et al., 2018 [40]	-	6	Over 80	Osteoporotic fracture or oblique inguinal hernia	0.20–186 ng/mL (cSf)			Glycosylated form of irisin is present in human cerebrospinal fluid. Irisin was not detected in plasma samples by using mass spectrometry.
Obesity								
Weber-Rajek et al., 2019 Poland [41]	Randomized control trial	49 (women)	67.00 ±6 (Control) 62.50 ± 2.0 (Experimental group)	Obesity	experimental group: 9.02 ± 2.67 control group: 5.91 ± 1.77			The authors observed a weak positive correlation between irisin and body mass index, however without statistical significance.
Sahin-Efe et al., 2018 USA [42]	Cross-sectional and a prospective case-control study	216	69.5 ± 9.2 (Non-obese normal fasting glucose) 66.9 ± 7.9 (Non-obese with impaired fasting glucose) 69.4 ± 8.6 (Obese with normal fasting glucose) 67.7 ± 7.4 (Obese with impaired fasting glucose)	Obesity	Non-obese normal fasting glucose: 123.6 ± 12.1 Non-obese with impaired fasting glucose: 124.8 ± 16.8 Obese with normal fasting glucose: 147.0 ± 16.2 Obese with impaired fasting glucose: 172.5 ± 13.0			Obese individuals with impaired fasting glucose have higher circulating irisin concentrations than non-obese subjects with normal glucose tolerance. Irisin concentrations do not predict the risk of developing diabetes prospectively.
Tibana et al., 2017 Brazil [43]	-	49 (women) Non-obese, n =23 Obese, n = 26	68.0 ± 6.2 (Non-obese) 66.5 ± 5.0 (Obese)	Obesity	Baseline Irisin concentration was 214.7 ± 53.2 ng Post-intervention mL for the non-obese and 225.0 ± 54.6 ng/mL for the obese group (184.1 ± 72.5 ng/mL; *p* = 0.011; 1 –ß = 0.95) with no change for the obese group (228.2 ± 59.5 ng/mL; *p* = 0.79)			No changes were observed in circulating irisin levels.
Sarcopenia								
Alsaawi et al., 2022 Saudi Arabia [44]	Cross-sectional study	131 (Women) Sarcopenia, n =26 No sarcopenia n = 131	65.9 ± 5.5	Sarcopenia	No sarcopenia: 180.8 ± 44.3 ng/L Sarcopenia: 145.8 ± 11.6 ng/L; *p* = 0.001			Irisin was significantly lower in the sarcopenia group. No associations were found with physical activity or dietary and lifestyle habits.
Baek et al., 2022 Republic of Korea [45]	Cross-sectional study	143 Sarcopenia, n =23	69.5 ± 6.16	Sarcopenia	Mean concentration 6.02 ± 1.46 ng/mL			Low irisin was associated with sarcopenia (OR = 0.97; 95% CI, 0.95–0.99; *p* = 0.002). No association was found between serum irisin levels and clinical muscle parameters.
Tsai et al., 2022 Turkey [46]	Not reported	72 No sarcopenia in older adults, n = 24 Sarcopenia in older adults, n = 24 Younger adults, n = 24	79.0 ± 5.9 (Older without sarcopenia) 79.4 ± 6.2 (Older adults with sarcopenia)	Sarcopenia	No sarcopenia 2.8 ng/mL (2.5, 3.2) Sarcopenia 3.1 ng/mL (2.2, 3.3)			No changes in irisin serum levels.
Park et al., 2019 Republic of Korea [47]		153 (women)	72.20 ± 5.96	Sarcopenia	Irisin was associated with sarcopenia (odds-ratio = 1.95, 95% confidence interval 1.33–2.87, *p*-value = 0.001)			In postmenopausal women, serum irisin may be used as a biomarker for sarcopenia.
Vascular disorders								
Bosanac et al., 2022 Slovenia [48]	Cross-sectional study	52	80.6 ± 6.6	Cardiovascular diseases	All (7.7) (3.5–19.5)	HFpEF with AF 4.8 (2.6–12.7)	HFpEF with AF 13.5 (7.1–31.5)	HFpEF and AF groups have significantly lower irisin levels compared to patients with HFpEF but without AF.
Tu et al., 2018 China [49]	Cross-sectional study	1530	66 (57–77)	Ischemic stroke	quartile 1 (<67.1 ng/mL), quartile 2 (67.1–87.8 ng/mL), quartile 3 (87.9–136.4 ng/mL), and quartile 4 (>136.4 ng/mL)			Irisin can be useful in predicting poor functional outcomes in ischemic patients.
Others								
Zhu et al., 2018 [50]	-	Control, n = 40 Cancer, n = 76	61.0 (59.0–66.0) (Control) 68.0 (62.0–76.0) (Cancer)	Colorectal cancer	Patient with colorectal cancer and normal weight: 0.17 ± 0.01 control 0.22 ± 0.01 μg/mL, *p* < 0.05			Individuals with high activating transcription factor 3 (ATF3) and low irisin levels were more likely to have colorectal cancer.

A/A—agitation/aggression; AF—atrial fibrillation; COPD—chronic obstructive pulmonary disease; CSF—cerebrospinal fluid; HFpEF—heart failure with preserved ejection fraction.

## Data Availability

Data can be accessed upon request to the authors.
